# The cumulative incidence and infection hospitalisation risk of SARS-CoV-2 by variant; a longitudinal study in England

**DOI:** 10.1093/aje/kwaf203

**Published:** 2026-01-08

**Keywords:** COVID-19, Cumulative incidence, infection-hospitalisation risk, MRP model

## Abstract

The COVID Infection Survey monitored daily positivity through the COVID-19 pandemic from 26-April-2020 to 13-March-2023. In total 451,079 participants in private residential households were enrolled in England and tested at regular intervals for SARS-CoV-2. Here, we estimated the cumulative incidence of PCR-positive infections using a multilevel regression and post-stratification model to obtain estimates of daily positivity, combined with a distribution of the duration of positivity from regular testing data. We estimated cumulative incidence by epoch (approximated by the dominance of successive SARS-CoV-2 variants) and calculated the corresponding infection-hospitalisation ratios. We found cumulative incidence was relatively low during pre-Alpha and Alpha-dominant epochs, rose steadily during the Delta-dominant epoch, and was highest during successive Omicron-dominant epochs. High cumulative incidences in successive Omicron-dominant epochs are consistent with lack of protection from previous infections. However, infection-hospitalisation ratios, whilst higher at the start of the pandemic, remained low after the Delta-dominant epoch and vaccine introduction. Stratified estimates show hospitalisation risk was consistently very low for younger age groups, increasing with age. Surveys with random sampling and longitudinal designs facilitate direct estimation of prevalence and incidence, however should be complemented by dense sampling to estimate duration of infection to maximise their value.

## Introduction

Since the first recorded case of SARS-CoV-2 in Wuhan (1), SARS-CoV-2 surveillance largely relied on data from national testing programmes (2,3). These are biased towards symptomatic presentation, which may vary across the stages of the pandemic. National testing data is also affected by the availability of testing and the cost and ease of access to testing. As these factors have also varied across the pandemic, applying a correction factor to national testing data to estimate the true number of infections in a population is problematic (4).

Alternatives sources of data on SARS-CoV-2 infections in the general population include community surveys, which can have either point prevalence (i.e. sampling different individuals in different survey waves) or longitudinal designs. In England, the REACT study is an example of the former (5), and the COVID-19 Infection Survey (CIS) (5) a UK-wide example of the latter (6). In CIS, a random sample of the population living in private residential households (including 451,079 people from England) were surveyed and tested at regular intervals from 26-April-2020 to 13-March-2023. CIS provides several advantages for estimating cumulative incidence. Similarly to REACT, the design allowed for detection of both symptomatic and asymptomatic infections, in contrast to many studies’ reliance on self-diagnosis of COVID-19 or self-presentation for testing (7)(8). Existing studies based on self-selection, i.e. with non-regular testing, have a bias towards detection of symptomatic individuals (8) thus tending to underestimate prevalence of SARS-CoV-2 and therefore underestimating incidence. Furthermore, symptomatic status is associated with viral clearance (9), therefore studies based on symptomatic population samples are likely to systematically overestimate the population viral clearance-time distribution - key to estimating incidence from prevalence data. Studies which rely on nucleocapsid antibody titres (4)(10), are unable to identify accurately when an individual was infected. In addition, antibody levels wane over time, underestimating cumulative incidence. While adjustments for waning can be made at a population level (11), these take time to develop.

In this study we have linked CIS to hospitalisation data (Hospitalisation Episode Statistics (HES)) allowing the number of hospitalisations from COVID-19 in the study population to be ascertained, time at risk calculated and hence community infection-hospitalisation risk estimated. This is important for understanding the impact of SARS-CoV-2 on healthcare resources and the risk posed by the infection to the population. Estimates from previous studies without individual level linkage (4) may have mismatches in sample demographics between the survey sample and publicly available health data. Furthermore, studies that underestimate SARS-CoV-2 cumulative incidence by not detecting asymptomatic infection thereby overestimate the risk of hospitalisation upon infection in the general population.

Reliable estimates over time of prevalence, viral clearance distribution, cumulative incidence, and hospitalisation risks are crucial to understanding the key drivers of the pandemic, as are how these change over time in reaction to public health and clinical interventions, changes in behaviour, and changes in the SARS-CoV-2 virus. This study provides these estimates, allowing assessment of the severity and trajectory of the pandemic over time, and the effectiveness of public health measures by epoch (approximated by dominance of a SARS-CoV-2 variant) and by age group.

## Methods

### Study Design

We used data collected by the Office for National Statistics COVID-19 Infection Survey between 26-April-2020 and 13-March-2023 (6). Private households were randomly selected from AddressBase, a commercially available address list (the majority; ~13% response rate), and previous surveys (initial recruitment only; ~50% response rates) on a continuous basis for enrolment from 26-April-2020 through 31-January-2022 (65% of participants were enrolled before December 2020, the start of the Alpha-dominant epoch).

Those in hospitals, care homes and other communal establishments were not included. Following verbal agreement to participate, a study worker visited each household to take written informed consent for individuals aged 2y and over. For those aged 2-15y, consent was provided by their parents or carers; those 10–15y also provided written assent. At the first visit, participants were asked for consent for optional follow-up assessments every week for the next month and then approximately monthly subsequently (>97% consented to monthly follow-up). The survey received ethical approval from the South Central Berkshire B Research Ethics Committee (20/SC/0195).

At each visit, participants answered survey questions and provided a nose and throat swab which was tested for SARS-CoV-2 using polymerase chain reaction (PCR) (additional details in appendix S1, S2) (parents/carers took swabs for children under 12y). Participants ≥16y in a random sub-sample of households were invited to provide blood for spike antibody testing. Home visits were conducted by study workers from 26-April-2020 to 31-July-2022; from 14-July 2022, home visits were replaced with online/telephone questionnaires and postal test kits and swabs returned by post or courier with no evidence of effect on swab positivity but slightly longer and broader distribution of time between assessments (12) (appendix S3, figure S1). Participants exited the survey if they moved home (since the household was sampled) and could also choose not to move to remote completion. Other than this, attrition rates (missing three consecutive visits) were <1%/month (appendix S3, figure s4)

This analysis included all participants from England (where linked hospital data was available) with at least one SARS-CoV-2 PCR test result in CIS from 26-April-2020 to 13-March-2023 (451,079 participants). We linked data from the CIS via the Personal Demographic Service (PDS) to Hospital Episode Statistics (HES) under the UK’s Digital Economy Act. The linkage rate between English CIS participants and HES was ~89%, with minimal linkage bias across demographic groups (appendix S4, figure S3).

## Statistical Methods

Our aims were two-fold, firstly to estimate the cumulative incidence of SARS-CoV-2 positivity by epoch (approximated as closely as possible to the dominance of a SARS-CoV-2 variant), and secondly to estimate the infection-hospitalisation risk by each corresponding epoch.

### Epochs

Analysis was based on periods of time (epochs) dominated by a SARS-CoV-2 variant, identified through S-gene target failure switching (as this was reliably associated with changes in dominant variant before November 2023) and supported by whole genome sequencing (13, 14). Epochs were based on surveillance weeks (starting on a Monday) and changed when S-gene presence/absence in CIS PCR tests with cycle threshold (Ct)<30 (where presence/absence can be reliably detected) switched above/below 50%, up to 7-November-2022, after which both S-positive (XBB* and CH.1.1*) and S-negative (BQ.1*) variants were circulating (appendix S5, figure S4 & S5). Specifically, the pre-Alpha epoch was before 06 December 2020) (S-positive), Alpha-dominant epoch from 07 December 2020 to 16 May 2021 (S-negative), Delta-dominant 17 May 2021 to 12 December 2021 (S-positive), Omicron BA.1-dominant 13 December 2021 to 20 February 2022 (S-negative), Omicron BA.2-dominant 21 February 2022 to 5 June 2022 (S-positive), and Omicron BA.4/5 dominant 6 June 2022 to 6 November 2022 (S-negative).

### Incidence

We define incidence as the number of people who would first test positive using a PCR test on a nose and throat swab each day, within an epoch, had they been tested, i.e. the number of new “infection episodes” per day. PCR test sensitivity and specificity were not adjusted for. We are unable to identify exactly when a participant was exposed to and first infected with SARS-CoV-2; our method may slightly overestimate cumulative incidence as the duration of infection would be longer if measured from exposure, as incidence is approximately prevalence divided by duration (15) (exact relationship below). As reinfections are both possible, and prevalent in Omicron epochs, the total number of new daily infections (cumulative incidence) does not equal the number of people ever infected.

To estimate how many people would have tested PCR-positive in each epoch, we estimated the number of people who would have tested positive on any given day (positivity) and then combined this with estimates of the duration of positivity (clearance) to obtain daily incidence. We then summed daily incidence by epoch to obtain cumulative incidence.

### Positivity

To estimate daily population-level positivity (appendix S6, figure S6), we used a Bayesian multilevel regression model with post stratification including all PCR test results across the entire time period (i.e. across all epochs together), and then predicted daily national positivity as a function of socio-demographic variables included in the model (age, sex, region) based on their distribution in the national population from Census 2021 (3)(6).

We used penalised complexity priors and a Laplace approximation to derive the posterior distribution, drawing 2000 subsequent samples. Time was modelled in days using a second order random walk and interacted with age group (2-11, 12-16, 17-24, 25-34, 35-49, 50-69, 70+ years). The model was estimated separately by region, so therefore included implicit interactions between region and time and region and age group. After post-stratification, the regions were weighted together (to account for regional population size) to obtain national estimates for England (appendix S6).

### Estimation of duration of PCR-positivity (clearance)

The analytical formula relating the incidence of new infection episodes to daily positivity (including repeat PCR-positives within the same infection episode) relies on an estimate of the duration of PCR-positivity within an infection episode. These “clearance times” (days from first becoming test-positive to first becoming test-negative) were estimated using CIS infection episodes between 01-September-2020 to 16-February-2023, defined using rules aiming to distinguish between reinfections and ongoing infections (appendix S2) (16). Without these rules, we may have wrongly classified reinfections as ongoing infections resulting in inflated clearance times. The “clearance times” were estimated as probability distributions using flexible proportional odds models for interval-censored data (Stata *stpm*), with the number and placement of knots determined by Akaike Information Criterion and subjective assessment to avoid over-fitting. Episodes were ‘right interval censored’ if a positive test was followed by a negative test, as the precise date on which the individual would have first tested negative is unknown; episodes could also be ‘right censored’, i.e. still positive at the last test. Calendar date was included as a natural cubic spline with one knot every two months to allow the clearance distribution to vary over calendar time, including both within and between epochs. The probability distribution of the duration of positivity is equal to one minus the distribution of time to clearance.

### Incidence of positivity

Incidence and duration of PCR-positivity (i.e. 1 minus clearance) can be used to estimate prevalence. The reverse process of estimating incidence from prevalence and duration of positivity is called “deconvolution” (15,17). Specifically, positivity on any given day is the sum of those testing positive on the current day and previous days who are still test-positive on the current day. A single linear equation relates prior (unknown) daily incidences, and corresponding (known) duration probabilities, to each day’s (known) positivity. Combining multiple days of positivity gives a system of linear equations which can be solved to give unknown daily incidences – though to solve the system, the number of unknowns (incidences) must be restricted by assuming known incidence values for the period before the prevalence series. However, if chosen reasonably, these assumed incidence values influence the subsequent incidence estimates only until clearance (of the assumed incident cases) is nearly complete – in practice less than 4 weeks. The deconvolution calculations were performed for each of the 2000 draws from the posterior positivity distribution from the multilevel model. We excluded the first few days of incidence estimates due to volatility, and present results from 1-May-2020. We estimated incidence in epochs where one SARS-CoV-2 variant dominated, assuming that reinfections with the same variant were rare. Of note, whilst positivity and incidence time series can be scaled and time-shifted to coincide (appendix S7, figure S7), the above formula, used in this study, is exact. The associated credible intervals reflect the uncertainty in the prevalence estimates but do not include uncertainty from the duration of positivity estimates or test sensitivity and specificity.

### Early incidence estimates obtained from spike antibody testing

CIS commenced on 26-April-2020, several months after the first UK COVID-19 case (29-January-2020). To obtain an overall estimate of the cumulative percentage infected from the start of the pandemic during the pre-Alpha epoch, we estimated the number of people infected prior to the survey’s start using the percentage of the random antibody sub-sample (n=3,298) tested between 26-April-2020 and 29-June-2020 positive for spike antibody (appendix S1). We weighted the antibody sample using population weights based on age, region and sex to obtain national level estimates. We did not adjust for sensitivity and specificity, however their effects are small (18) given high test specificity and sensitivity before widespread vaccination. We used data until 29-June-2020 to increase power, and allow for those infected by 26-April-2020 to develop detectable antibodies.

The overall estimate for the pre-Alpha period therefore combined this static antibody-based estimate of the number of individuals infected by 1 May-2020 together with the modelled cumulative incidence estimate from 1-May-2020 to 7-Dec-2020. The latter was estimated exactly as described above but subtracted from the post-stratification denominator the number of people that S-antibody prevalence suggested had already been infected before the start of the survey, to avoid double-counting.

### Estimating infection-hospitalisation risk

We included only hospital admissions with COVID-19 recorded as the primary cause (primary ICD-10 diagnostic code of U07.1 or U07.2). Hospitalisation data was not available for the last epoch. For each CIS participant, we calculated time at risk by epoch; defining the entry date as the maximum of enrolment and the epoch start date, and the exit date as the minimum of the last assessment date, the epoch end date, and the first hospitalisation date in that epoch. For each epoch, we calculated time at risk and enumerated first COVID-19 admissions for each CIS participant, then derived the hospitalisation rate with confidence intervals (19) (appendix S4) and then used the following formula which also accounted for any residual unrepresentativeness of survey participants compared with the national population

*Primary hospitalisations per day = (Hospitalisation rate per 100*,000 person-days *English population)/100,*000*

Hospitalisations per epoch = Primary hospitalisations per day*Length of epoch (days)

*Infection-hospitalisation risk per epoch = Hospitalisations per epoch/Cumulative incidence estimate per epoch (i.e. the total number of estimated infections)*.

### Sensitivity analysis

Sensitivity analyses tested the effect of varying distributions for the time to clearance, namely the probability distribution of the number of days from the first test-positive until a participant first tests negative (i.e., the duration of PCR-positivity); the availability of publicly available daily data from the National Basketball Association (NBA) study (9) meant we were able to identify when a participant changed from positive to negative without interval censoring in the Delta-dominant and Omicron-dominant epochs. We estimated time from first positive to first negative test using Kaplan-Meier methods, after data cleaning following the authors’ guidance (9) and excluding the small number of participants with only one test result. Persistent positives were right censored. The NBA study recruited a convenience sample including team staff, players, arena staff, vendors, and others affiliated with the NBA; 66.6% of test results in the study were in those >31y (9).

Analyses were conducted in R version 4.1.0 and STATA version 16.1.

## Results

**Figure 1:**
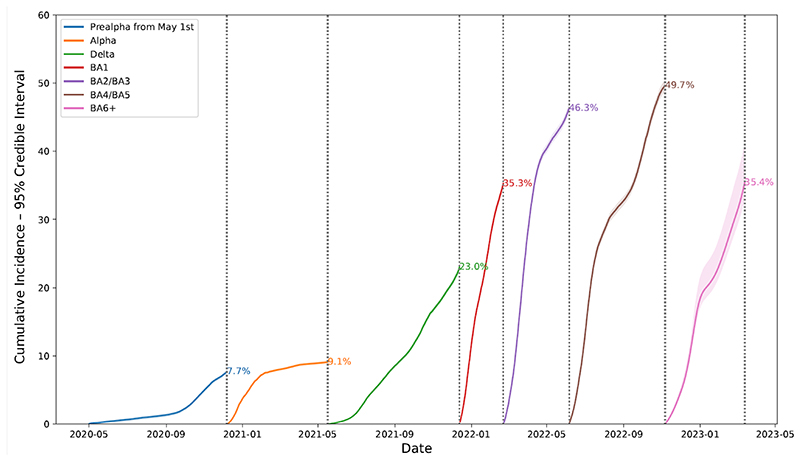
Estimated cumulative incidence of SARS-CoV-2 PCR-positivity from May 1^st^ 2020 by epoch, England Note: from spike antibody testing 6.3% of the English population had been infected with SARS-CoV-2 by 1-May-2020.

**Figure 2:**
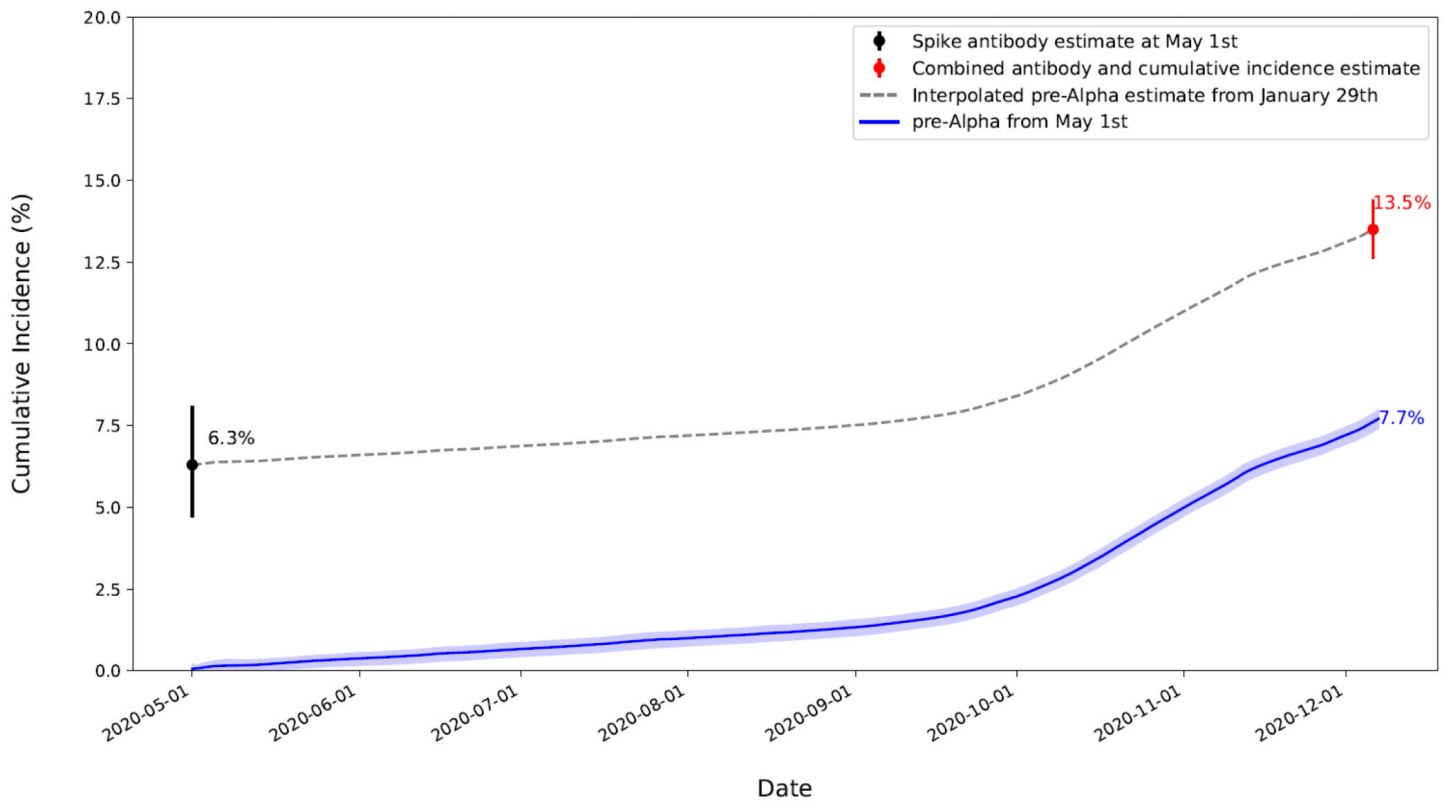
Estimated spike antibody estimate and cumulative incidence of SARS-CoV-2 during the pre-Alpha epoch, England. Note: To avoid double counting, those suggested as infected prior to May 1^st^ based on antibody test were removed from the denominator to obtain the combined estimate. As a result, the estimates from the antibody and cumulative incidence estimate are not exactly equal to our combined estimate.

Figure 1 shows estimated cumulative incidence in England by epochs defined by the dominance of successive SARS-CoV-2 variants (and hence not comparable in duration). CIS began on 26-April-2020; we therefore did not directly observe the first few months of SARS-CoV-2 infections. However, as shown in Figure 2 estimates from spike antibody testing suggested that approximately 6.3% (95% confidence interval (CI) 4.7-8.1%) were infected by 1-May-2020 (16). Combining this antibody estimate with the cumulative incidence estimate (see Methods) suggested that 13.5%, 95% credible interval (CrI) 12.6-14.4%) of the population had COVID-19 by 06-December-2020. Over the period directly included in our survey (1-May-2020 to 06-December-2020) we estimated 7.7% (95% CrI 7.4-8.0%) cumulative incidence, compared to 9.1% (9.0-9.3%) for the Alpha-dominant epoch (07-December-2020 to 16-May-2021), 23.0% (22.7-23.3%) for Delta-dominant (17-May-2021 to 13-December-2021), 35.3% (34.7-35.8%) for BA.1-dominant (14-December-2021 to 21 February 2022), 46.3% (45.7-47.0%) for BA.2-dominant (22-February-2022 to 5-June-2022), 49.7% (48.8-50.4%) for BA.4/5-dominant (06-June-2022 to 06-Nov-2022), and 29.6% (25.0-34.6%) subsequently (07-November-2022 to 13-March-2023). The relatively high incidence of reinfections post-Omicron means these percentages cannot be summed to get a total percentage of the population ever infected.

**Figure 3:**
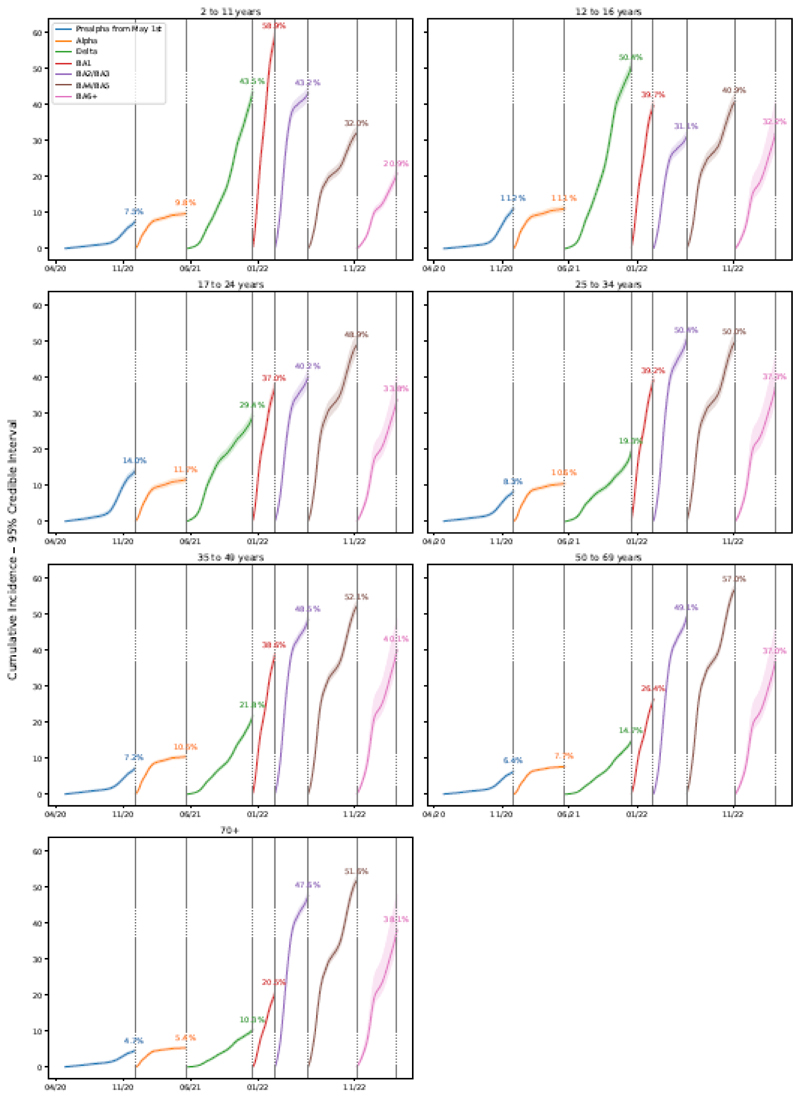
Estimated cumulative incidence of SARS-CoV-2 PCR-positivity from May 1^st^ 2020 by epoch, stratified by age group, England

Figure 3 shows estimates by age from 01-May-2020. From 01-May-2020 and during the Alpha-dominant epoch, cumulative incidence was generally low across all age groups, likely at least partly due to the severity of government restrictions. The cumulative incidence for those aged 70+ years was particularly low in both Pre-Alpha (from 01-May-2020) and Alpha-dominant epochs at 4.7% (95% CrI 4.3-5.0%) and 5.4% (5.1-5.7%) respectively. Amongst the youngest age groups (02-11, 12-26, 17-24 years) consistently high cumulative incidence was seen from Delta-dominant epochs onwards, implying a high level of reinfections across epochs.

### Sensitivity analysis

**Figure 4:**
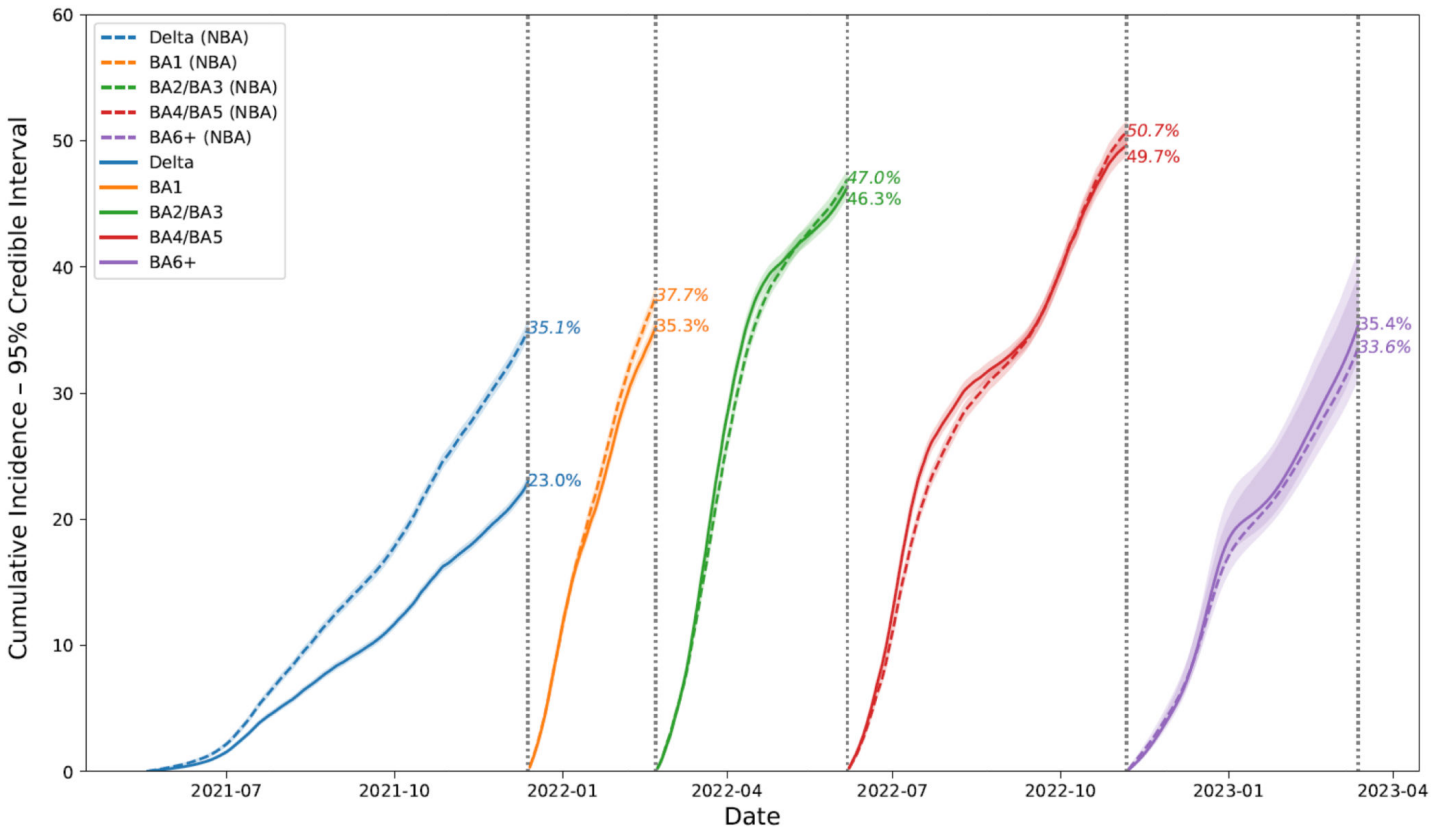
Estimated cumulative incidence of SARS-CoV-2 PCR-positivity by epoch using different distributions for the distribution of the duration of PCR-positivity (shown in parentheses), England

For the Delta-dominant epoch, using the NBA clearance distribution resulted in a higher cumulative incidence of 35.1% (95% CrI 34.6-35.7%) versus 23.0% (22.7-23.3%) using CIS distributions (Figure 4). However, the NBA Delta estimate was based on only 238 participants with positive tests. For the Omicron-dominant epochs, the differences in cumulative incidence were negligible. No pre-Delta data were available from the NBA study.

### Infection-Hospitalisation Risk

The infection-hospitalisation risk was, as expected, higher early in the pandemic before the introduction of widespread vaccination. Stratified estimates by age (table 1) showed the hospitalisation risk was consistently very low for those 2-34y at 0.21 % (0.11-0.30%) in the Alpha-dominant epoch, dropping further with Delta to 0.10% (0.07-0.14%). Similarly for those aged 35-64 the risk was low during Alpha at 1.44% (1.23-1.66%) and fell further with Delta to 0.46% (0.38-0.54%). Those 65y+ had notably higher hospitalisation risks at 6.98% (5.50-8.46%) during pre-Alpha and 4.51% (3.84-5.17%) during the Alpha-dominant epoch, with the risk dropping during the Delta-dominant epoch to 1.94% (1.64-2.23%) following vaccine introduction. The risk across all groups was low during successive Omicron-dominant epochs.

## Discussion

Here, we have considered the estimation of cumulative incidence of SARS-CoV-2. Our approach is to reasonably assume few re-infections within an epoch dominated by one variant, so all infections within an epoch are assumed to be first infections with that variant. Cumulative incidence can then be reasonably interpreted as the percentage of people infected during each epoch.

We previously released a national estimate of cumulative incidence in England from 27-April-2020 to 11-February-2022 using a variation of the method detailed here (20). Originally, we only included positive tests in first infection episodes to estimate daily positivity. However, as noted, the first infection episode observed in our data may not truly be the first; participants may have been infected before enrolment, or previously during their participation but not tested PCR-positive. With increasing reinfections following the emergence of Omicron variants, this method was not sustainable: the method employed here largely circumvents the problem of reinfections by providing estimates by epoch. Our estimates of cumulative incidence are broadly consistent with those modelled by research groups using alternative data sources and different methods (21) (22) and with our initial approaches using CIS data (23) (appendix S8, figure S8).

Over the pandemic, reinfections became increasingly common, although remained relatively low with the same variant (13). Excepting the first pre-Alpha and the last study period (from 7-November-2022), strain replacement happened very quickly (appendix S5, figure S4 & S5) (13), meaning that epochs could be defined by dominant strains and there was relatively little strain diversity within epochs other than the first and last. We have previously shown that only infection with the current or most recent prior variant provided any protection against reinfection, protection which also waned over time (4). Together this means we can confidently estimate cumulative incidence within each epoch (13) (14), but not across epochs. To accurately estimate the percentage of people who have ever had COVID-19 across all periods, we need an estimate of the number of re-infections over time, which is challenging.

The corresponding hospitalisation-infection ratios demonstrate the waning of the risk of severe disease posed to the general population over time, likely due to a combination of factors. Many studies reported that the risk of hospitalisation following vaccination rollout measurably diminished (24–26); correspondingly we found lower hospitalisation-infection rates during Delta- and Omicron-dominant epochs. It is plausible that the “at-risk” pool of people in the population reduced naturally owing to increasing immunity from prior infections (27). However, the cumulative incidence was relatively low for both pre-Alpha and Alpha-dominant epochs. More plausibly, the reduction in infection-hospitalisation risk was predominantly due to vaccination rather than infection-related immunity, and specific characteristics of the Omicron variants (28,29). Differing hospitalisation rates across the Omicron epochs may be due to factors such as characteristics of our survey population (e.g. higher uptake of third booster vaccinations during BA.1) and availability of non-hospital treatments (antivirals and monoclonal antibodies) at symptom onset in vulnerable individuals in later epochs which were not available earlier in the pandemic.

### Limitations

This study exploits a unique data collection effort; considering the limitations and their impact may strengthen similar data collection efforts in future pandemics. We derived cumulative incidence by combining estimates of the duration of PCR-positivity and daily positivity from a random sample of the population, the latter post-stratified to account for residual lack of representativeness of the survey sample. Clearly people’s behaviours may differ, with survey respondents potentially more likely to practice behaviours which reduced transmission. However ultimately an infectious pathogen circulating at high levels in society means the impact of individual behaviours can only limit to a certain extent the risk of transmission; especially for children, who would be exposed via schools etc.

Our survey did not include care homes and communal establishments; as these consist of a relatively small proportion of the population, their exclusion is unlikely to have had a measurable impact on the cumulative incidence had they been included. However, omitting care homes does mean the infection-hospitalisation risk would unlikely be applicable to the entire population, as care home residents are particularly vulnerable to severe illness (30), Studies using publicly available hospitalisation data which included the very frail and care home residents reported higher infection hospitalisation risk (31).

The main limitation methodologically is identifying how long individuals are PCR-positive (clearance), with various estimates in the literature (9) (31,32). Here, we estimated the clearance distribution using PCR tests from the survey for primary analyses. Whilst some participants were on weekly visits (predominantly in the pre-Alpha and Alpha epochs), we can be reasonably confident in accuracy; however, as the survey progressed most participants were on monthly visits and the time between visits became slightly longer. Our estimates were designed to account for missed infections falling between tests; however, if the duration of PCR-positivity shortened over time and we missed more infections, we have less observed data on which to base estimates, possibly impact the accuracy of the estimated clearance distribution. As a surveillance study testing participants independently of symptoms, a substantial proportion of infections were identified “late” with high Ct values; further, the percentage of late infections decreases when incidence is increasing and vice versa, leading to an epidemic phase bias (33). This is problematic because the number of infections missed likely varies over time, and this variation may not be fully captured by our clearance distribution. Overall, our sensitivity analysis using the more selected but more densely sampled NBA population, suggests that the CIS clearance distribution provides similar inference for Omicron-dominant epochs, but has greater differences for the Delta-dominant epoch. However, in the Delta-dominant epoch, the NBA sample size was smaller and participants could join the study whilst positive, reducing reliability of these estimates. A consideration for future studies would be a joint Bayesian analysis incorporating existing clearance estimates into a duration prior.

An additional major limitation is that we have not incorporated the uncertainty from our duration of positivity estimates into our incidence estimates; the credible intervals around incidence reflect the uncertainty of the positivity estimates and likely underrepresent the true uncertainty. However, using bootstrap to estimate a range of clearance distributions in our original work (20) and then combining these with the Bayesian draws had minimal impact on uncertainty in incidence estimates; however given this limitation the uncertainity measures should be interpreted with some caution, acknowledging the additional aspects of uncertainty not fully captured. Furthermore, we calculated the incidence of PCR-positive infection not exposure, we do not know when an individual was first exposed to SARS-CoV-2, and as the time between exposure and test positivity may vary across the pandemic, we cannot simply apply a correction factor (4). Finally, we did not adjust for PCR sensitivity and specificity; previous analysis suggests specificity was close to 99%. Our data cannot inform about test sensitivity without providing a very informative prior on the true prevalence (6) (12).

This analysis is based on periods of time (epochs) dominated by a SARS-CoV-2 variant, as we were unable to sequence every PCR-positive due to high Ct/low viral load in a substantial minority, and similarly when Ct values are high S-gene target failure is not a reliable proxy. This is more problematic at the beginning of each epoch, as we do not know whether an infection is with the previous dominant variant, or the new variant, although positivity rates were generally low at this time (35). Some infections will have been caused by variants that never dominated, meaning that epoch and variant are not fully interchangeable.

### Surveillance of future pandemics

The United Kingdom invested heavily in monitoring COVID-19, funding several large studies. In parallel to CIS, the ZOE Covid Study monitored infection cases via a smart phone app in volunteers (36) and REACT-1 (5) and REACT-2 (10) monitored infections in repeated point prevalence surveys of individuals randomly selected from primary care practice lists via PCR tests and antibodies respectively. Resultantly, key public policy decisions were based on several high quality estimates. The combination of a randomly selected sample and continuous testing was an advantage of CIS, enabling continuous estimate of positivity over time, in contrast to the extrapolation between survey waves required by the design of REACT (37).

As above, our most challenging limitation was estimating infection duration, which was challenging with the design of REACT (38) or Zoe. A future option to consider is more frequent testing amongst a sub-sample of participants, or following an initial positive PCR test. However relevant considerations include 1) increased cost 2) burden to participants of high frequency testing (leading to higher dropout) 3) size of the sample required, especially at times of low prevalence 4) logistics of additional testing after an initial positive given delays in receiving results and sending out tests. Nevertheless, this would reasonably accurately estimate infection duration and possibly provide information on infectiousness.

Notwithstanding the limitations, the cumulative incidence of PCR-positive infection is a robust estimate enumerated on the private residential population in England. This coupled with the accompanying infection-hospitalisation risk demonstrates the breadth and severity of the pandemic overtime and the pressures on health services.

## Supplementary Material

Supplementary Materials

## Figures and Tables

**Table 1:** Infection-hospitalisation ratios by epoch and age, England **Age 2-34**

Epoch	Number in CIS	Person- Days at risk	Primary hospitalisations over epoch	Infection-hospitalisation ratio % (95% CI)*
Pre Alpha-dominant (01 May - 07 December 2020)	93935	7306757	**	-
Alpha-dominant (08 December 2020 - 17 May 2021)	106397	15204546	21	***0.21 (0.11-0.30)***
Delta-dominant (18 May 2021 – 13 December 2021)	113382	20398980	33	***0.10 (0.07-0.14)***
BA1-dominant (14 December 2021 – 21 February 2022)	100277	6794296	25	***0.06 (0.03-0.8)***
BA2-dominant (22 February 2021 – 06 June 2022)	96660	9574167	21	***0.05 (0.03-0.08)***
BA4/5-dominant (07 June 2022 – 06 November 2022)	85695	11901761	17	***0.05 (0.02-0.08)***

* Infection-hospitalisation ratio per epoch = Derived hospitalisations per epoch/Cumulative incidence estimate per epoch

** Numbers suppressed due to low counts

**Table:** Age 35-64

Epoch	Number in CIS	Person-Days at risk	Primary hospitalisations over epoch	Infection-hospitalisation ratio % (95% CI)*
Pre Alpha-dominant (01 May - 07 December 2020)	142255	11469119	63	***1.84 (1.36-2.32)_***
Alpha-dominant (08 December 2020 - 17 May 2021)	163668	24051870	193	***1.44 (1.23-1.66)***
Delta-dominant (18 May 2021 – 13 December 2021)	182117	34172373	133	***0.46 (0.38-0.54)***
BA1-dominant (14 December 2021 – 21 February 2022)	171440	11722812	81	***0.11 (0.08-0.13)***
BA2-dominant (22 February 2021 – 06 June 2022)	168449	17057976	99	***0.12 (0.10 - 0.15)***
BA4/5-dominant (07 June 2022 – 06 November 2022)	157174	22386586	93	***0.12 (0.09-0.14)***

* Infection-hospitalisation ratio per epoch = Derived hospitalisations per epoch/Cumulative incidence estimate per epoch

**Table:** Age 65 +

Epoch	Number in CIS	Person- Days at risk	Primary hospitalisations over epoch	Infection-hospitalisation ratio % (95% CI)*
Pre Alpha-dominant (01 May - 07 December 2020)	81704	6889004	100	***6.98 (5.50-8.46)***
Alpha-dominant (08 December 2020 - 17 May 2021)	91095	13558562	205	***4.51 (3.84-5.17)***
Delta-dominant (18 May 2021 – 13 December 2021)	103947	19468687	185	***1.94 (1.64-2.23)***
BAl-dominant (14 December 2021 – 21 February 2022)	98324	6733871	62	***0.31 (0.23-0.40)***
BA2-dominant (22 February 2021 – 06 June 2022)	97108	9897405	145	***0.32 (0.27-0.38)***
BA4/5-dominant (07 June 2022 – 06 November 2022)	92054	12804106	174	***0.41 (0.34-0.47)***

* Infection-hospitalisation ratio per epoch = Derived hospitalisations per epoch/Cumulative incidence estimate per epoch
