# Supplementary material for: The cumulative incidence and infection hospitalisation risk of SARS-CoV-2 by variant; a longitudinal study in England
Source: Am J Epidemiol. Author manuscript; Available in PMC 2026 Mar 14. (PMC7618863; doi:10.1093/aje/kwaf203)
Supplement: Supplementary Materials [file EMS212847-supplement-Supplementary_Materials.docx]

**Supplementary material**

**The cumulative incidence and infection hospitalisation risk of SARS-CoV-2 by variant; a longitudinal study in England**

Charlotte Gaughan , David Braunholtz , Leanne Massie , Tarnjit Khera, Paul J Birrell, Daniela De Angelis, Josh Blake, Joy Preece, Koen Pouwels, Ann Sarah Walker, the COVID-19 Infection Survey Team

**S1: Swab Information**

Swab positivity

Antibody positivity

**S2: Infection episodes**

**S3: Representativeness of survey**

Number of participants in survey (table S1)

Participants and visits over time (figure S1, figure S2)

**S4: CIS population linked to Hospitalisation Data**  (figure S3)

**S5: Definition of epoch** (figure S4, figure S5)

**S6: Model Specification**

MRP model

Positivity over time (figure S6)

**S7: Positivity vs Incidence** (figure S7)

**S8: Cumulative Incidence Estimate Comparison** (figure S8)

***S1 - Swab Information***

Nose and throat self-swabs were couriered directly to the UK's national Lighthouse laboratories (National Biocentre in Milton Keynes and Glasgow) where samples were tested as part of the UK’s national testing programme. Identical methodology was used to test for the presence of SARS-CoV-2 genes for nucleocapsid protein (N), spike protein (S), and ORF1ab using RT-PCR. We used the TaqPath RT-PCR COVID-19 kit (Thermo Fisher Scientific, Waltham, MA, USA), which was analysed using UgenTec Fast Finder 3.300.5 (TagMan 2019-nCoV assay kit V2 UK NHS ABI 7500 v2.1; UgenTec, Hasselt, Belgium) (1). The assay plugin contains an assay-specific algorithm and decision mechanism that allows conversion of the qualitative amplification assay PCR raw data from the ABI 7500 Fast into test results with little manual intervention. Samples are called positive in the presence of at least one gene (N, ORF1ab, or both) but could be accompanied by the gene for S protein (i.e. one, two, or three gene positives) (1). The gene for S protein is not considered a reliable single gene positive (2).

Blood samples were couriered to the clinical biochemistry and microbiology laboratories at the John Radcliffe Hospital in Oxford to test for the presence of antibodies using the Thermofisher immunoassay (2) (3). Normalised results were reported in ng/ml of mAb45 monoclonal antibody equivalents. Before 26 February 2021, the assay used fluorescence detection as described previously, with a positivity threshold of 8 million units (42 ng/ml) validated on banks of known SARS-CoV-2 positive and SARS-CoV-2 negative samples (3) (4). After this, it used a commercialized CE-marked version of the assay, the OmniPATH 384 Combi SARSCoV-2 IgG ELISA (Thermo Fisher Scientific), with the same antigen and colorimetric detection (1).

***S2 - Definition of Infection Episodes***

We first grouped repeated positive tests from the Covid Infection Survey into infection episodes, which were then used in all analyses. To reflect the fact that some individuals test positive on PCR for extended periods of time when testing is independent of symptoms/case contacts as in this study (in contrast to national testing programmes), whereas others have reinfections (confirmed by sequencing) after only short periods of time, we incorporated information from genetic sequencing, S-gene presence/absence, and cycle threshold (Ct) values, together with negative PCR test results from CIS tests. Using criteria developed through expert consensus, careful inspection and analysis of CIS data alone, and considering definitions of reinfection used elsewhere (5-8), we defined the start of a new infection episode as any of the following: 1) a new swab positive occurring >120 days after an index positive with the preceding test being negative, based on analyses of vaccine effectiveness against Delta and Alpha variants which showed that definitions based on shorter periods of time and/or without a previous negative in these earlier calendar periods erroneously included those testing PCR-positive for long periods of time (9) or 2) >90 days with the preceding two consecutive tests being negative (one negative after 20 December 2021 when Omicron variants dominated given higher re-infection rates with Omicron (5) (10), or 3) >60 days with the three preceding consecutive tests being negative, or 4) after 4 preceding consecutive negative test results at any time.

## We then split these infection episodes if they had grouped together positive tests containing multiple sequences from different genetic lineages (e.g. BA.5 and BA.2), or had incompatible S-gene target positivity consistent with co-circulating variants with Ct<30 (e.g. S-gene positive and S-gene negative, both with Ct<30 during periods when BA.1 and BA.2 were co-circulating), or had large decreases in Ct or low Ct long after the first positive within an episode (both indicative of a new infection rather than ongoing PCR positivity).

***S3 - Representativeness of the COVID-19 Infection Survey***

**Table S1: Number of survey participants with at least one positive or negative PCR test by socio demographic characteristics**

| **Characteristic** | **Number of Participants** | **Participants (%)** | **Census (%)** |
| --- | --- | --- | --- |
| Cohort size (England) | 451,079 |  |  |
| Sex female | 238,507 | 52.9 | 51.0 |
| Sex male | 212,572 | 47.1 | 49.0 |
| Age group 2-11 | 35,504 | 7.9 | 12.0 |
| Age group 12-16 | 24,842 | 5.5 | 6.0 |
| Age group 17-24 | 27,452 | 6.0 | 9.7 |
| Age group 25-34 | 48,169 | 10.7 | 13.7 |
| Age group 35-49 | 90,982 | 20.2 | 19.9 |
| Age group 50-69 | 147,286 | 32.7 | 24.8 |
| Age group 70+ | 76,884 | 17.0 | 13.8 |
| Ever health condition yes | 107,082 | 23.7 | - |
| Ever health condition no | 343,997 | 76.2 | - |
| Ethnicity White | 411,154 | 91.1 | 81.0 |
| Ethnicity Asian | 21,049 | 4.7 | 9.6 |
| Ethnicity Black | 5,264 | 1.2 | 4.2 |
| Ethnicity Mixed | 8,950 | 2.0 | 3.0 |
| Ethnicity Other | 4,662 | 1.03 | 2.2 |
| Region North East England | 19,107 | 4.2 | 4.7 |
| Region North West England | 60,520 | 13.4 | 13.1 |
| Region Yorkshire and the Humber | 42,696 | 9.5 | 9.7 |
| Region East Midlands | 34,048 | 7.6 | 8.6 |
| Region West Midlands | 39,896 | 8.9 | 10.5 |
| Region East England | 50,339 | 11.2 | 11.2 |
| Region London | 93,725 | 20.8 | 15.6 |
| Region South East | 67,669 | 15.0 | 16.4 |
| Region South West | 43,079 | 9.6 | 10.1 |

***Participants and visits over time***

A total of 467295 participants across the UK contributed to the survey through study worker home visits before 31 July 2022. Among those who remained in the survey in May 2022 and were therefore invited to move to remote data collection (N=338463), 324903 participants (96%) chose to continue (11).

The distribution of the intervals between study assessments is presented below (Figure S1). The overall median duration between study assessments was 28 days (IQR 26-35). Most of the intervals between assessments were <45 days (11). Noting that initial recruitment was on a rolling basis, the distribution of the number of tests per participant in the survey over time is presented in Figure S2.

**Figure S1 - Distribution of the intervals between study assessments**


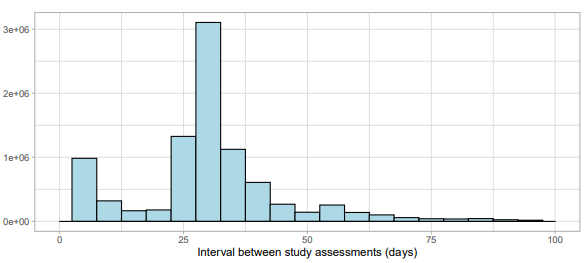


**Figure S2 - Distribution of the number of assessments per participant in the survey over time**


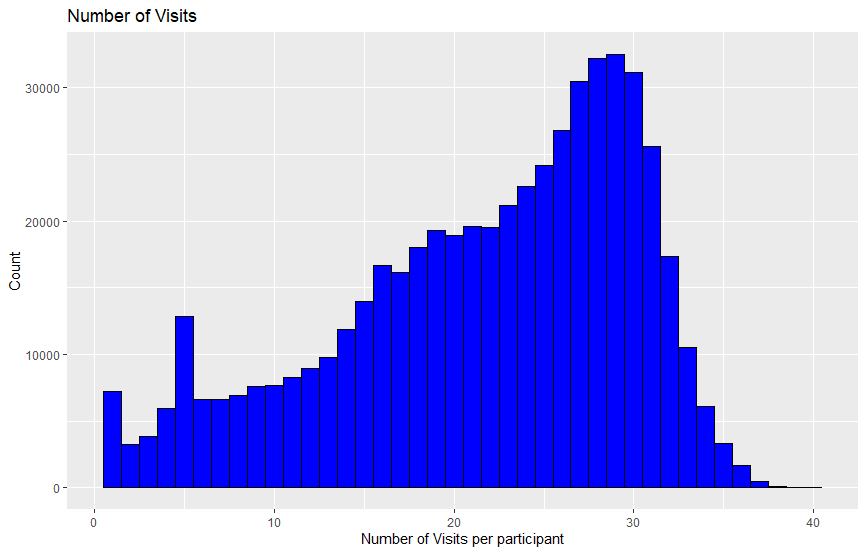


***S4*. *Characteristics of CIS population who could be linked to hospitalisation data***

In CIS, 252,309 (47.1%) English participants were male and 283,328 (52.9%) were female. In the CIS-HES linked dataset this was 228,217 (47.2%) and 255,215 (52.7%) respectively.

In CIS, 422,100 (78.8%) English participants never reported having a long-term health condition, 96,060 (17.9%) reported having a long-term health condition and 17,480 (3.3%) responses were missing. In the CIS-HES linked dataset 380,520 (78.7%) participants never reported having a long-term health condition, 85,970 (17.8%) reported having a long-term health condition and 16,940 (3.5%) responses were missing.

The mean age for all English CIS participants was 47.7 years, with a median age of 51.0 years. The mean age of the CIS-HES linked participants linked was 47.6 years, with a median age of 50.0 years.

***Figure S3 - Distribution of ages for all English CIS participants (left) and CIS participants linked to HES (right)***


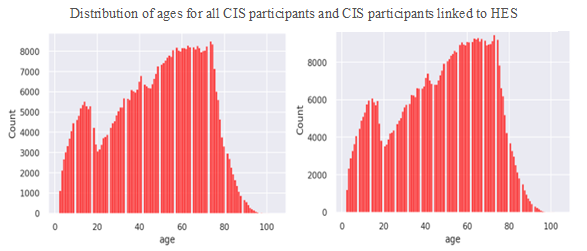


**Confidence intervals for infection-hospitalisation ratio**

The confidence intervals for the infection-hospitalisation ratio were approximated as (12)*:*


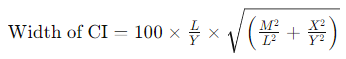

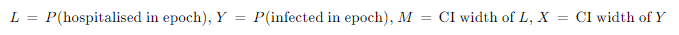


***S5. Definition of epochs where variants dominated***

The PCR test used in the survey included the S-gene as a target, meaning we were able to track the emergence of new variants using S-gene target failure (Figures S3, S4). Excepting the first pre-Alpha and last epochs studied, there was relatively little genetic diversity within epochs where individual variants dominated (>85% prevalence, indicated by horizontal lines). Epochs were therefore defined by switch in prevalence between across 50% which happened when absolute positivity was low (13)

**Figure S4 - S-positivity in all CIS positives**

**Figure S5 – S-positivity in CIS positives with cycle threshold (Ct)<30**

_

***S6. Model specification***

*MRP model*

To estimate positivity, we used a multilevel regression model with post-stratification. A Laplace approximation was used to estimate the posterior probability distribution of the model paramters and positivity (14).

The model was fitted at a regional level. The unit of observation was an individual swab/test result; in particular, we assumed that there were n swabs taken, and the i-th of these was associated with time ti, and a vector of other covariates xi. The probability of the i-th swab being positive is then given by a generalised linear model.

We included sex as a fixed effect with 2 levels, while age group (7 levels) was included using random effects. Time was included in days using a second order random walk.

This model was implemented using the following R syntax using the R-INLA package (https://www.r-inla.org/documentation):

Result ~ 1 + sex + f(ageg_small, model =”iid”, hyper = list( prec = list (prior = “pc.prec”), param = c(1,0.1) + f(daynr, model = “rw2”, hyper = list (prec = list (prior = “pc.prec”, param = c(1,0.1) + f( ageg_small, model = “iid”, group= daynr, control.group =list(model = “rw2”)

*Where:*

*ageg_small = age groups 2-11, 12-16, 17-24, 25-34, 35-49, 50-69, 70+ years*

*daynr = day number*

**Model for the probability of the i-th swab being positive**


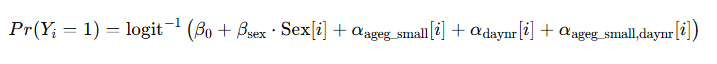


**Model notation :**


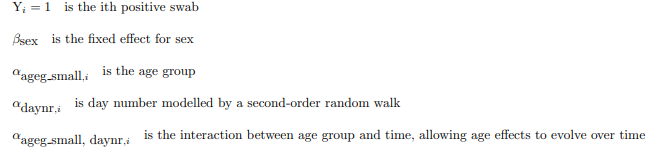


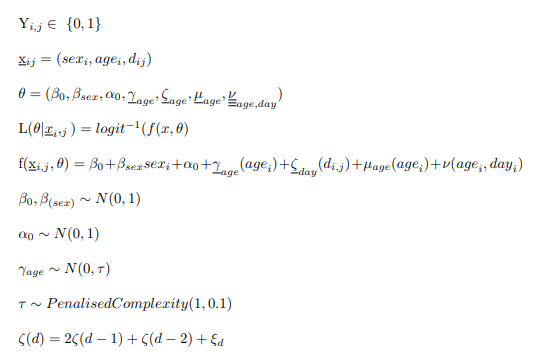


**Post stratification**


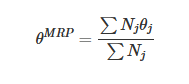


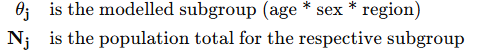


***Figure S6 – SARS-CoV-2 positivity by age groups over time in England***


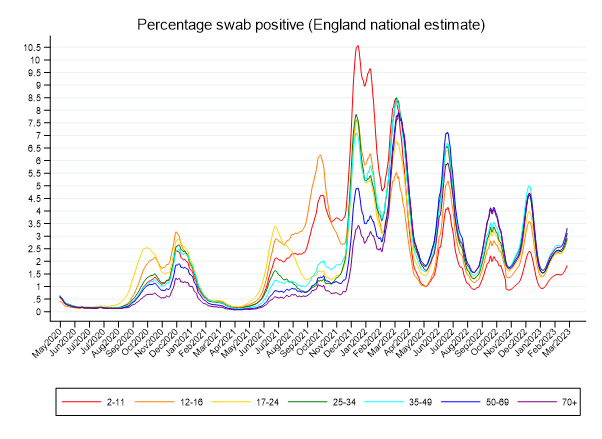


***S7. Positivity vs Incidence***

As described in the main Methods, there is a direct analytical relationship between incidence and prevalence, via integration of the product of incidence and duration of infection. However, incidence and prevalence estimates can also be time-shifted and scaled for comparison. On average over the course of pandemic, prevalence was roughly 8.5 times greater than positivity, noting that the duration of positivity estimates vary across the pandemic. Estimating the maximum cross correlation suggested an average lag of approximately 6 days between incidence and prevalence.

***Figure S7 – Positivity over time compared with incidence (scaled)***


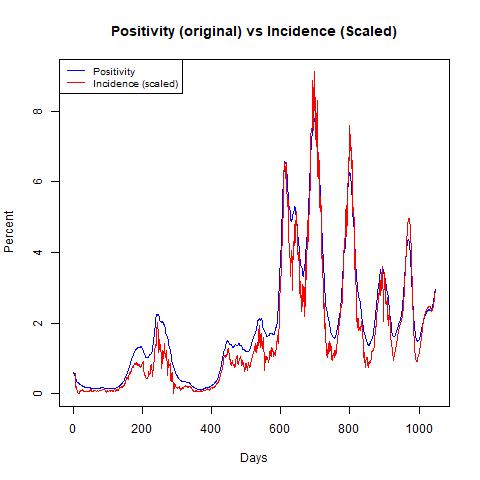


***S8*. *Covid Infection Study (ONS) Cumulative Incidence Estimate Comparison*** (15)

The methodology presented in the paper, which presents cumulative incidence by epoch, builds on a previous attempt to estimate cumulative incidence across the pandemic. Due to increasing reinfections, which became increasingly prevalanet with Omciron, we switched to estimating by epoch.

This figure presents our earlier publicly available estimates (ONS Estimate) to produce cumulative incidence estimates up to Omicron, compared to REACT-2 and a separate published modelled estimated using publically available cases (18)

**Figure S8 – Comparison of cumulative incidence estimates across studies**


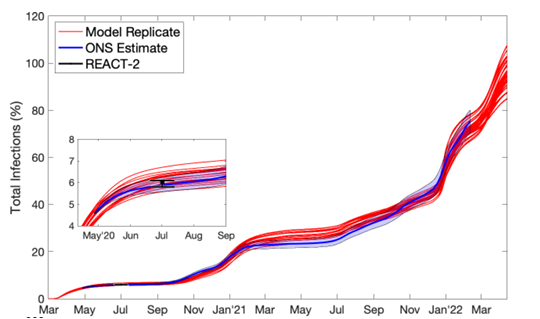


***References***

1. Pouwels, K.B. et al. Improving the representativeness of UK’s national COVID-19 infection Survey through spatio-temporal regression and post-stratification. Nature Communications. 2024: 15; 5340
2. Pouwels, K.B. et al. Community prevalence of SARS-CoV-2 in England from April to November, 2020: results from the ONS Coronavirus Infection Survey. Lancet Public Health, 2021; e30-e38
3. National SARS-CoV-2 Serology Assay Evaluation Group. Performance characteristics of five immunoassays for SARS-CoV-2: a head-to-head benchmark comparison. Lancet Infectious Disease. 2020: 1390-1400 .
4. Wei, J. et al. Antibody response and correlates of protection in the general population after two doses of the ChAdOx1 or BNT162b2 vaccines. Nature Medicine. 2022, 28, 1072-1082.
5. Pulliam, J. R. C. et al. Increased risk of SARS-CoV-2 reinfection associated with emergence of Omicron in South Africa. Science. 2022: 376
6. Bowe, B., Xie, Y. & Al-Aly, Z. Acute and postacute sequelae associated with SARS-CoV-2 reinfection. Nature Medicine. 2022. 28:11 28, 2398–2405 .
7. Chemaitelly, H. et al. Immune Imprinting and Protection against Repeat Reinfection with SARS-CoV-2. New England Journal of Medicine 2022: 387, 1716–1718
8. Yahav, D. et al. Definitions for coronavirus disease 2019 reinfection, relapse and PCR re-positivity. Clinical Microbiology and Infection 2021, 315–318: 27
9. Pritchard, E., Matthews, P.C., Stoesser, N. et al. Impact of vaccination on new SARS-CoV-2 infections in the United Kingdom. Nat Med 27, 2021: 1370–1378.
10. Nguyen, N. N. et al. High rate of reinfection with the SARS-CoV-2 Omicron variant. Journal of Infection 2022: 85:174–211
11. Wei, J., Stoesser, N., Matthews, P.C. *et al.* Risk of SARS-CoV-2 reinfection during multiple Omicron variant waves in the UK general population. *Nat Commun* **15**, 1008 (2024).
12. Díaz-Francés et al. On the existence of a normal approximation to the distribution of the ratio of two independent normal random variables, Statistical Papers, 2012, 54 (2). Springer Science and Business Media LLC: 309–323.
13. Pritchard E, Vihta KD, Eyre DW, Hopkins S, Peto TEA, Matthews PC, Stoesser N, Studley R, Rourke E, Diamond I, Pouwels KB, Walker AS, Infection Survey Team C. Detecting changes in population trends in infection surveillance using community SARS-CoV-2 prevalence as an exemplar. Am J Epidemiol. 2024 Dec 2;193(12):1848-1860
14. Rue H, Riebler A, Sørbye SH, Illian JB, Simpson DP, Lindgren FK. Bayesian computing with INLA: a review. Annual Review of Statistics and Its Application . 2017, 4: 395-421
15. Keeling MJ. Patterns of reported infection and reinfection of SARS-CoV-2, Journal of Theoretical Biology, 2023, 556, 111299
